# Explosion Detection Using Smartphones: Ensemble Learning with the Smartphone High-Explosive Audio Recordings Dataset and the ESC-50 Dataset

**DOI:** 10.3390/s24206688

**Published:** 2024-10-17

**Authors:** Samuel K. Takazawa, Sarah K. Popenhagen, Luis A. Ocampo Giraldo, Jay D. Hix, Scott J. Thompson, David L. Chichester, Cleat P. Zeiler, Milton A. Garcés

**Affiliations:** 1 Infrasound Laboratory, Hawaiʻi Institute of Geophysics and Planetology, School of Ocean and Earth Science and Technology, University of Hawaiʻi at Mānoa, Kailua-Kona, HI 96740, USA; spopen@hawaii.edu (S.K.P.); milton@isla.hawaii.edu (M.A.G.); 2 Idaho National Laboratory, Idaho Falls, ID 83415, USA; luis.ocampogiraldo@inl.gov (L.A.O.G.); jay.hix@inl.gov (J.D.H.); scott.thompson@inl.gov (S.J.T.); david.chichester@inl.gov (D.L.C.); 3 Nevada National Security Site, North Las Vegas, NV 89030, USA; zeilercp@nv.doe.gov

**Keywords:** explosion, smartphone, machine learning, detection, data, infrasound

## Abstract

Explosion monitoring is performed by infrasound and seismoacoustic sensor networks that are distributed globally, regionally, and locally. However, these networks are unevenly and sparsely distributed, especially at the local scale, as maintaining and deploying networks is costly. With increasing interest in smaller-yield explosions, the need for more dense networks has increased. To address this issue, we propose using smartphone sensors for explosion detection as they are cost-effective and easy to deploy. Although there are studies using smartphone sensors for explosion detection, the field is still in its infancy and new technologies need to be developed. We applied a machine learning model for explosion detection using smartphone microphones. The data used were from the Smartphone High-explosive Audio Recordings Dataset (SHAReD), a collection of 326 waveforms from 70 high-explosive (HE) events recorded on smartphones, and the ESC-50 dataset, a benchmarking dataset commonly used for environmental sound classification. Two machine learning models were trained and combined into an ensemble model for explosion detection. The resulting ensemble model classified audio signals as either “explosion”, “ambient”, or “other” with true positive rates (recall) greater than 96% for all three categories.

## 1. Introduction

Explosions generate infrasonic (<20 Hz) and/or low-frequency sounds (<300 Hz) that can travel vast distances. The travel distance and frequency range of these sounds depend on the size of the explosion and atmospheric conditions. For reference, the peak central frequency of a pressure wave from a 1 ton of trinitrotoluene (TNT) explosion would be around 6.3 Hz, and it would be around 63 Hz for a 1 kg TNT explosion [1]. This phenomenon can and has been used to detect explosions. For example, the International Monitoring System (IMS) has a network of globally distributed infrasound sensors to detect large (>1 kiloton) explosion events [2]. Similarly, for smaller-yield explosions, there have been examples of infrasound and seismoacoustic sensors deployed on regional and local scales [3,4,5,6,7,8,9]. However, as networks become denser, the cost and difficulty of covering a wider area grows rapidly. Thus, many of these networks are deployed temporarily for experiments or around specific areas of interest, such as volcanos or testing sites [10], and optimized for the prevailing weather patterns.

With the unfortunate rise in global tension, there has been increased interest in smaller-yield explosions, especially the potential for targeted attacks at locations such as ports, power plants, and populated places [11,12]. In such cases, the prompt detection of smaller-yield explosions in key locations and regions could be crucial as fast and reliable detection would lead to decreased response times and could potentially reduce casualties and damage. However, as mentioned previously, having a dense sensor network for low-yield explosions becomes expensive and difficult to maintain. A solution to this problem is using non-traditional sensors such as smartphones, especially considering recent advancements and success in mobile crowd sensing [13,14,15]. The ability of smartphones to capture acoustic and accelerometric explosion signals comparable to those captured by traditional sensors has been shown in previous studies [10,16], and machine learning methods have been used to determine the range and intensity of four explosions from 52 accelerometer and pressure signals collected by smartphones [17,18]. The results of these studies provide strong evidence that using smartphones is a viable solution. However, further progress on detection and classification models is limited by the small volume of publicly available data. Thus, we have released a labeled collection of 326 multi-modal data from 70 explosions to the public [19], and, in this work, demonstrate the ability of machine learning methods to detect explosion signals recorded on smartphones.

The release of the Smartphone High-explosive Audio Recordings Dataset (SHAReD) [19], the labeled data that we collected on smartphone networks, provides a unique dataset that can be utilized for machine learning (ML) methods for explosion detection. The audio data from the high-explosive (HE) dataset were used in conjunction with data from an external environmental sound dataset (ESC-50 [20]) to train two separate machine learning models, one using transfer learning (YAMNet [21]) and the other considering only the low-frequency content of the waveforms. These two models were then combined into an ensemble model to classify audio data as “explosion”, “ambient”, or “other”. Although the two models both performed well while classifying the sounds individually, each model had its own shortcomings in distinguishing between the categories. We found that by combining the two models into one ensemble model, the strengths of each model compensated for the shortcomings of the other, significantly improving performance.

### Transfer Learning, YAMNet, and Ensemble Learning

Transfer learning (TL) is a machine learning technique which utilizes a pre-trained model as a starting point for a new model designed to perform a similar task. TL has gained popularity as it can compensate for the consequences of having a limited amount of data on which to train a model [22]. TL using convolutional neural networks (CNNs), such as Google’s Yet Another Mobile Network (YAMNet), has become common practice for environmental sound classification [23,24,25,26].

**Figure 1:**
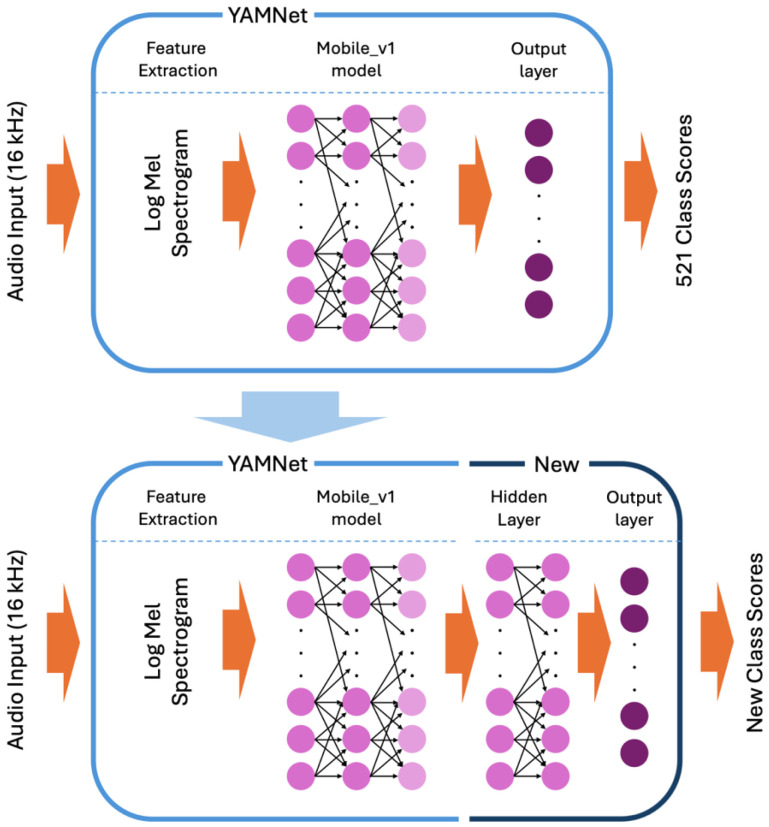
The architecture of YAMNet and an example architecture of a transfer learning model using YAMNet.

YAMNet is an off-the-shelf machine learning model trained on data from AudioSet [27], a dataset of over 2 million annotated YouTube audio clips, to predict 521 audio classes [21]. It utilizes the Mobile_v1 architecture, which is based on depth-wise separable convolutions [28]. The model can take any length of audio data with a sample rate of 16 kHz. However, the data are split internally into 0.96 s frames with a hop of 0.48 s. This is carried out by taking the full audio data to compute a stabilized log Mel spectrogram with a frequency range of 125 Hz to 7500 Hz and dividing the results into 0.96 s frames. These frames are then used in the Mobile_v1 model, which produces 1024 embeddings (the averaged-pooled output of the Mobile_v1 model). These embeddings are fed into a final output layer that produces the 521 audio classes’ scores. For TL, the final output layer is removed, and the embeddings are used to train a new model, as seen in Figure 1.

Ensemble learning (EL) is a machine learning technique which produces a prediction by utilizing the predictions of multiple trained models. This combined prediction generally performs better than any single model used in the ensemble by compensating for individual models’ biases and by reducing overfitting [29]. In the context of ESC, since there are numerous types of environmental sounds, it would be beneficial to have multiple “expert” models that are trained on specific signals rather than a single all-encompassing model [30]. There are multiple ways to implement EL, such as by combining the results of multiple similar models trained on different subsets of the data (“bagging”) or by training a model on the predictions of different ML models trained on the same data (“stacking”) [31]. In this study, we trained two different models on the same data and determined the final prediction based on pre-defined criteria that will be discussed later.

## 2. Data and Methods

### 2.1. Smartphone High-Explosive Audio Recordings Dataset (SHAReD)

**Figure 2:**
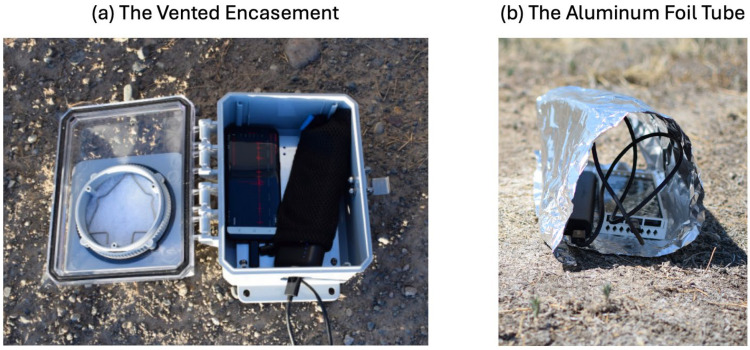
The smartphone deployment configuration of (**a**) the vented encasement and (**b**) the aluminum foil tube.

SHAReD consists of 326 multi-sensor smartphone data from 70 surface HE events collected at either Idaho National Laboratory or Nevada National Security Site [19]. The RedVox application [32] was used to collect and store the smartphone data, which consisted of data from the microphone, accelerometer, barometer, Global Navigational Satellite System (GNSS) location sensor, and other metadata such as the smartphone model and the sample rate of the sensors. For a more comprehensive description of the RedVox application, we direct the reader to Garcés et al., 2022 [32]. The smartphones were deployed at varying distances near the explosion source in a vented encasement or aluminum foil tube alongside an external battery, as shown in Figure 2. The different deployment configurations were used to protect the smartphones from the elements, specifically direct sunlight, which can cause overheating, or precipitation, which can damage the internal circuitry.

**Figure 3:**
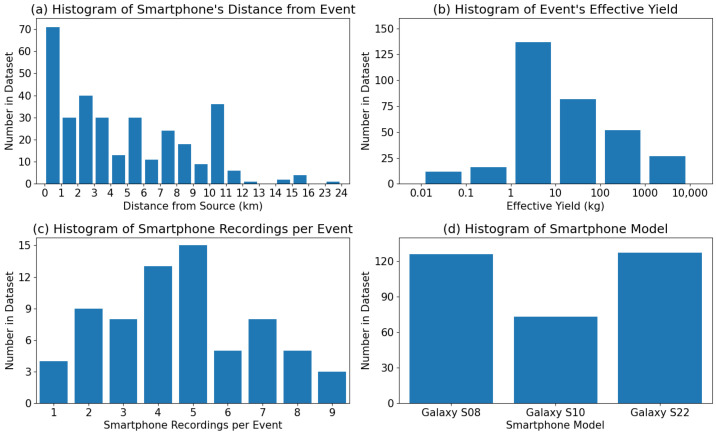
Histograms of (**a**) the smartphone’s distance from the explosion source, (**b**) the effective yield of the explosion source, (**c**) the number of smartphone recordings per explosion event, and (**d**) the smartphone model used for data collection in SHAReD.

The distances of the smartphones from the explosion source ranged from around 430 m to just over 23 km, but the majority were within 5 km, as seen in Figure 3a. Most of the explosions had effective yields (the amount of TNT required to produce the same amount of energy in free air) between 1 and 100 kg, and the total range spanned from 10 g to 4 tons, as seen in Figure 3b. Due to differing organizational policies surrounding individual events, the true yield of each explosion may or may not be included in the dataset. However, the effective yield range is included for all events. The histogram of the number of smartphone recordings per event is shown in Figure 3c. Although there were at least five deployed smartphones for each event, we see that there were a few dozen events with less than five smartphone recordings included in the dataset. This discrepancy is caused by either the yield of the explosion being too small for the signal to travel to all the smartphones or the atmospheric conditions of that day adding significant noise to the signal, as any smartphone recordings with signal-to-noise ratios of 3 or less were removed from the dataset. The smartphones used for the dataset were all Samsung Galaxy models, either S8, S10, or S22. The dataset spans multiple years, and the smartphones used for the collection were periodically replaced with newer models. The overall distribution of smartphone models represented in the dataset can be seen in Figure 3d. Further details about the explosion data will be included in a later section.

As previously mentioned, the time-series data included in the dataset are from the microphone, accelerometer, barometer, and the GNSS location sensor. However, the extracted explosion signals from all the sensors were based on the acoustic arrival as the name of the dataset suggests. The duration of the extracted signal is 0.96 s and contains the acoustic explosion pulse. Although each set of smartphone data contains a clear explosion signal in the microphone data, depending on the distance of the phone and yield of the explosion, there may not be a visible signal for the accelerometer or barometer data. This was in part due to the higher sensitivity and sample rate of the smartphone microphone sensor. For reference, the sample rate for the microphone was either 800 Hz (63 recordings) or 8000 Hz (263 recordings), whereas the sample rates for the accelerometer averaged around 412 Hz and those of the barometer averaged around 27 Hz. Additionally, 0.96 s of “ambient” audio data was included in the dataset by taking microphone data from before or after the explosion. Overall, the smartphone microphones captured a filtered explosion pulse due to their diminishing frequency response in the infrasonic range; however, the frequency and time–frequency representations showed great similarities to explosion waveforms captured on infrasound microphones. We direct those interested in further information on explosion signals captured on smartphone sensors and/or how they compare to infrasound microphones to the work of Takazawa et al., 2024b [10], in which a subset of SHAReD is used.

### 2.2. Training Data

In addition to the explosion and ambient microphone data from SHAReD, audio from the ESC-50 dataset was also used to train the ML models. This was carried out to increase the robustness of the model as, in uncontrolled environments (i.e., urban areas), the recorded data are not limited to explosions or ambient sounds and can include a broad spectrum of frequencies [33,34]. Additionally, previous work showed improvement due to the inclusion of the ESC-50 dataset by reducing false positive classifications of non-explosion sounds [35]. The ESC-50 dataset is a collection of 2000 environmental sound recordings from 50 different classes, and it is often used for benchmarking environmental sound classification [25,36,37]. The audio classes can be separated into 5 broad categories of animal, nature, human, domestic, and urban. Some examples of the classes include keyboard typing, clapping, cow and sounds that contain infrasound such as thunderstorm and fireworks. Each class contains 40 sets of 5 s clips recorded at a sample rate of 44.1 kHz.

Since there were differences in the data (i.e., sample rate, duration, recording instrument), some standardization methods were applied to prepare for machine learning. First, the ESC-50 waveforms were trimmed to a duration of 0.96 s to match the waveforms in SHAReD. Trimming was conducted by taking a randomized segment of the waveform that contained the maximum amplitude. The randomization was added to avoid centering the waveforms on a peak amplitude that could be used as a false feature of ESC-50 waveforms that the ML models could learn. Secondly, the waveforms’ sample rates were adjusted to create two separate datasets with constant sample rates for each of the two ML models. The sample rates were standardized by upsampling or downsampling the waveforms. Thirdly, labels were applied to each category of waveforms (explosion recordings labeled “explosion”, ambient recordings labeled “ambient”, and recordings from ESC-50 labeled “other”). Lastly, the dataset was randomly split into 3 sets: the training set, the validation set, and the test set. Since there was an imbalance in the amount of data (326 each for “explosion” and “ambient”, 2000 for “other”), the split was applied for each label to ensure a balanced distribution of data (stratified splitting). Additionally, the “explosion” and “ambient” data were split by the explosion event to ensure that the ML models would be robust by testing the model on data from explosions that it had not seen. Since grouping the explosion events can cause an imbalance in the characteristics of explosion data (i.e. yield), the random selection had a check to ensure the characteristic distribution between the training and test set was within 10%. The dataset was roughly split into 60%, 20%, and 20% for the training, validation, and test sets.

### 2.3. Machine Learning Models

The first model, called Detonation-YAMNet (D-YAMNet), was trained using TL with the YAMNet model. The primary reason for using TL was to compensate for the small dataset (<3000). D-YAMNet was constructed by replacing the final output layer of YAMNet with a fully connected layer containing 32 nodes with rectified linear unit (ReLU) activiation and an output layer with 3 nodes corresponding to “ambient”, “explosion”, and “other”. Sparse categorical cross-entropy was used for the loss function and Adamax [38] was used for the optimizer. In order to further mitigate overfitting, the final number of nodes was chosen by iterating through different values during training and selecting the smallest value that kept a minimum of 90% precision for each category when classifying the validation set. The number of epochs was set to 400; however, early stopping by monitoring the validation accuracy was implemented to avoid overfitting. Additionally, class weights were added to address the imbalance in the amount of data in the “ambient” and “explosion” categories compared to the “other” category.

The second model was designed to complement the D-YAMNet model by focusing on the lower-frequency components of these waveforms since the YAMNet architecture drops all frequency content below 125 Hz. This model will be referred to as the low-frequency model (LFM). To ensure the model concentrated on the low-frequency portion of the input waveform, the sample rate was limited to 800 Hz. Although numerous arguments can be made for different architectures for the LFM, we chose a compact 1D CNN as it is well-suited for real-time and low-cost application (i.e., smartphones) and has shown greater performance on applications that have labeled datasets of limited size [39]. The LFM consisted of a 1D CNN layer with 16 filters and a kernel size of 11 and ReLU activation, followed by a 50% dropout layer, a max pooling layer with pool size of 2, a fully connected layer with 32 nodes with ReLU activation, and a 3-node output layer. The dropout layer and max pooling layers were added to mitigate overfitting since the LFM is trained on a limited dataset. Additionally, like D-YAMNet, the specifics (number of filters, kernel size, and number of nodes) of the model were determined through iteration and selection of the least complex values that kept a minimum of 90% precision for each category. The same loss function, optimizer, epoch numbers, early stopping, and class weights were used for the training of the LFM as for D-YAMNet.

**Figure 4:**
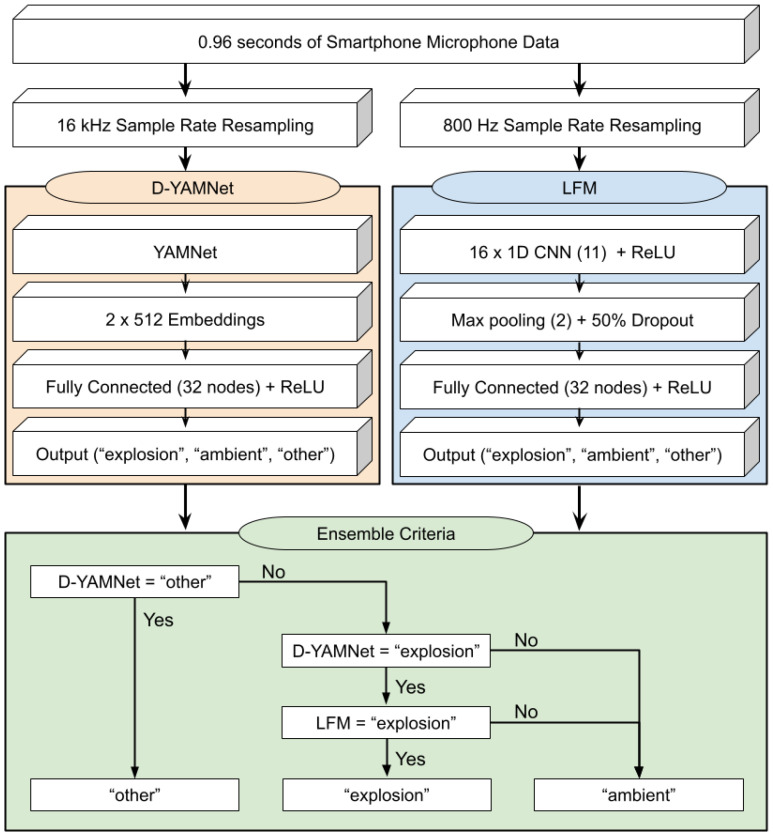
Flowchart for the ensemble model along with the construction of D-YAMNet and LFM models.

The ensemble model was constructed using the predictions from the D-YAMNet and LFM with the following criteria: “explosion” if both models predicted “explosion”, “other” if D-YAMNet predicted “other”, and “ambient” for all other cases. For the ease of the reader, the flowchart for the ensemble model along with the model construction for D-YAMNet and LFM are presented in Figure 4. For EL using two separate models, stacking is generally used. However, we chose these criteria based on the overall purpose of the ensemble model and insight from the construction of the two incorporated models. The “explosion” prediction was only selected if both models predicted “explosion” to reduce false positive cases, as they can pose an issue for continuous monitoring. The “other” category was solely based on the D-YAMNet prediction since it has the added benefits of TL and covers a wider frequency range that many “other” sound sources would fall under. Although accurately predicting if a non-explosion signal is in the “other” category is not the primary goal for the model, it plays a crucial role in reducing false positive cases for “explosion” as it creates a category for other sounds that a smartphone may pick up while deployed. Overall, these criteria allow the ensemble model to essentially use the LFM to assist D-YAMNet in determining whether a waveform classified as “explosion” by the latter should be classified as “explosion” or “ambient”.

## 3. Results

The models were evaluated using the test set, which was about 20% of the whole dataset and included explosion events that were not included in the training or validation set. The results are showcased using a normalized confusion matrix, where the diagonal represents each category’s true positive rate (recall). The confusion matrix was normalized to clarify results, given the imbalance in the dataset.

### 3.1. D-YAMNet

**Figure 5:**
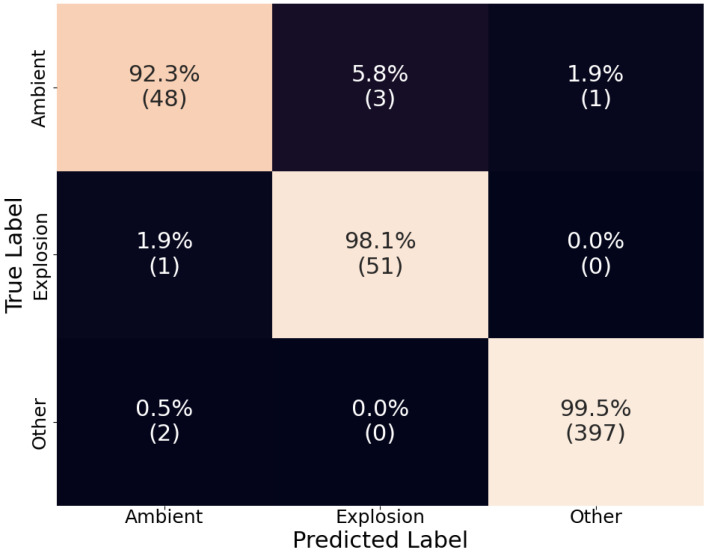
The confusion matrix of D-YAMNet on the test dataset. The percentages are calculated by rows (true labels) and the count for each cell is listed under these in parenthesis.

Overall, D-YAMNet performed well, with each category’s true positive rates of 92.3%, 98.1%, and 99.5% for “ambient”, “explosion”, and “other”, respectively (Figure 5). The model especially performed well in the “other” category. Although, this model’s purpose is not to identify “other” sound events, this category was added to reduce false positive “explosion” predictions and was successful as there were no false positive cases. In contrast, the model performed worse in the “ambient” category. This relatively low recall of the “ambient” category is most likely due to the nature of the model.

**Figure 6:**
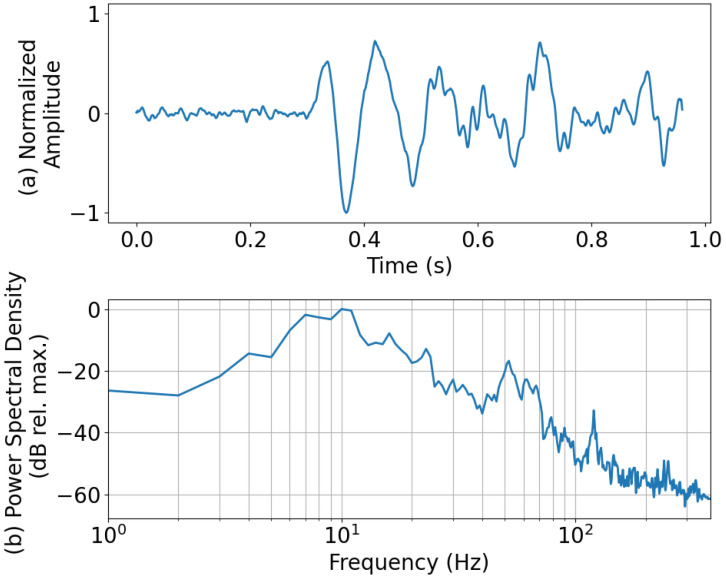
The (**a**) normalized amplitude and (**b**) power spectral density of an “explosion” waveform that was misclassified as “ambient” by D-YAMNet. The explosion was in the 10 kg yield category and recorded by a smartphone ~11 km away from the source at a sample rate of 800 Hz.

As described earlier, YAMNet ignores frequencies that are below 125 Hz, which is where most of the energy of the explosion signal lies. This could make distinguishing between “explosion” and “ambient” difficult, especially if the waveforms lack significantly identifiable higher-frequency content. To illustrate this, the “explosion” waveform that was falsely categorized as “ambient” is presented along with its power spectral density in Figure 6. This misclassified “explosion” waveform was from an explosion in the 10 kg yield category and recorded on a smartphone roughly 11 km from the source at a sample rate of 800 Hz. For reference, the probability of each class taken from the SoftMax layer of the D-YAMNet was 0.696, 0.304, and 0.000 for “ambient”, “explosion”, and “other”, respectively. From an initial glance at the normalized amplitude (Figure 6a), we see that the waveform was heavily distorted. Looking at the power spectral density (Figure 6b), most of the frequency content of the waveform was concentrated below 100 Hz. Additionally, the small frequency spike seen past the 100 Hz mark was located at 120 Hz, which is below the 125 Hz cutoff of the YAMNet model. This majority of the signal’s energy concentration being below the YAMNet’s frequency cutoff, paired with the lack of higher frequency content due to the 800 Hz sample rate of the smartphone microphone, is most likely what led to the model misclassifying the explosion as “ambient”.

### 3.2. Low-Frequency Model

**Figure 7:**
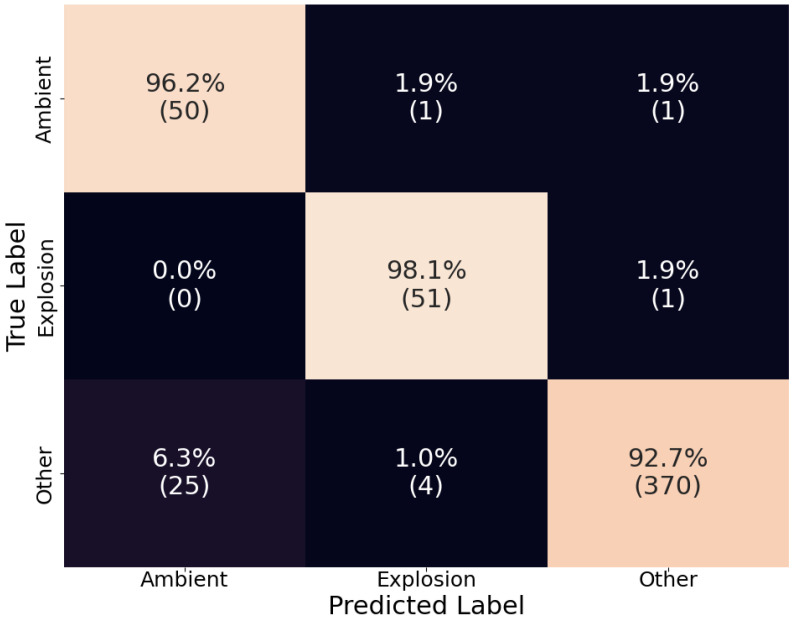
The confusion matrix of the LFM on the test dataset. The percentages are calculated by rows (true labels) and the count for each cell is listed under these in parenthesis.

Unlike D-YAMNet, the LFM incorporates all the frequency content of the input data. However, since the waveforms used for training were downsampled to 800 Hz, the model was not trained on the higher-frequency content of the signals. This resulted in a somewhat reversed outcome compared to D-YAMNet, as seen in Figure 7. Overall, the LFM performed worse than D-YAMNet, which was expected since it was trained on a small dataset without the benefits of transfer learning.

**Figure 8:**
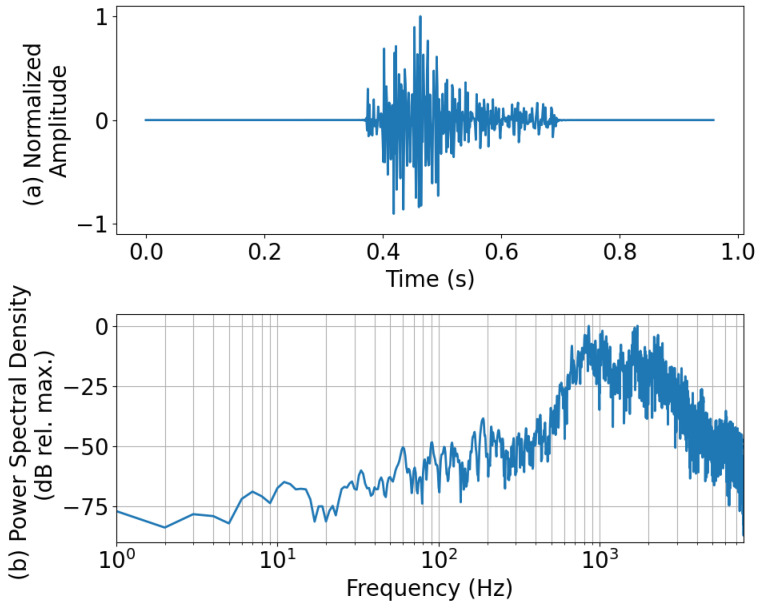
The (**a**) normalized amplitude and the (**b**) power spectral density of an “other” waveform that was misclassified as “explosion” by the LFM. The “other” sound was from an ESC-50 waveform labeled “dog”.

Looking at the recall scores and comparing them to those from D-YAMNet (Figure 5), we see that the “ambient” category performed better (96.2%), “explosion” performed the same (98.1%), and “other” performed worse (92.7%). The relatively high recall scores for the “ambient” and “explosion” classes are likely due to the low-frequency content of the waveforms being kept. However, the worse performance in the “other” category is most likely due to misclassification of those ESC-50 data that mostly contain higher-frequency content, which would be removed in the downsampling process, making them indistinguishable from “ambient” or “explosion” waveforms. As an example of the latter, a waveform from the “other” category that was misclassified as an “explosion” is presented along with its power spectral density in Figure 8. This “other” waveform was labeled “dog” in the ESC-50 dataset. The probabilities for each class taken from the SoftMax layer of the LFM for this waveform were 0.346, 0.514, and 0.140 for “ambient”, “explosion”, and “other”, respectively. Looking at that the normalized amplitude (Figure 8a), we see that it was a transient sound; however, it does not necessarily resemble an explosion pulse to an experienced eye. Moving to the power spectral density (Figure 8b), most of the frequency content of the waveform was concentrated above 500 Hz, which is below the 400 Hz cutoff (Nyquist) of the LFM. Additionally, there was a decent amount of energy for frequencies down to 60 Hz. The majority of waveforms’ frequency content being above the cutoff for LFM while also containing some energy in the lower frequencies is most likely what led to the model misclassifying the waveform as “explosion”, although the associated probability was low (0.514).

### 3.3. Ensemble Model

**Figure 9:**
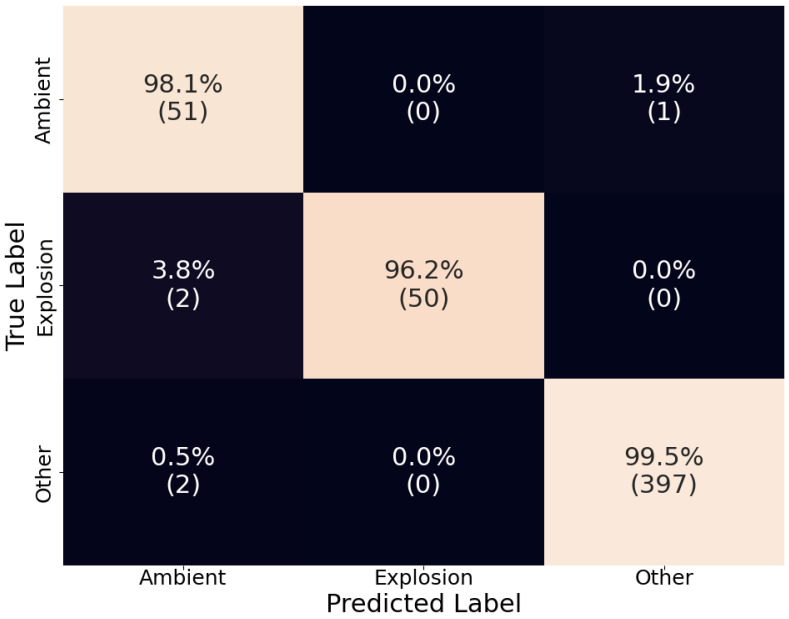
The confusion matrix of the ensemble model on the test dataset. The percentages are calculated by rows (true labels) and the count for each cell is listed under these in parenthesis.

The confusion matrix for the ensemble model can be seen in Figure 9. Altogether, we see an increased performance with recall above 96% for each category. Only the recall of the “explosion” category saw a slight decrease due to the inclusion of false negatives from both D-YAMNet and LFM. Focusing on the “ambient” and “other” categories, we see that the proposed criteria preserved the high performance of D-YAMNet in the “other” category, while, simultaneously, the addition of the LFM improved the results in the “ambient” category. More importantly, we see that the ensemble model was able to eliminate false positives in the “explosion” category entirely, which translates to a much more stable and robust model that can be used for real-time explosion monitoring.

## 4. Discussion and Conclusions

Two ML models (D-YAMNet and LFM) were trained and tested using two datasets (SHAReD and ESC-50) and then combined into an ensemble model for explosion detection using smartphones. Although both D-YAMNet and LFM had recall scores over 90% for each category, each model had a weak point in one category: “ambient” for D-YAMNet and “other” for LFM. These shortcomings were explained by the construction of the models. For D-YAMNet, the low-frequency information (<125 Hz) of the waveforms was not incorporated, which would make it difficult to distinguish “ambient” sounds (which were mostly silent) from “explosion” sounds, which have the majority of their energy concentrated in the lower frequencies. For LFM, the sample rate of the input data was limited to 800 Hz, which made “other” sounds with primarily high-frequency information harder to distinguish from “ambient” sounds. The ensemble model was able to compensate for each model’s shortcomings, which resulted in a combined model that performed better overall, with >96% recall in each category. Additionally, the ensemble model eliminated all false positive cases in the “explosion” category, creating a more robust model for explosion detection.

### 4.1. Precision-Recall Curves of D-YAMNet and LFM 

For binary classification, receiver operating characteristic (ROC) curve is commonly used for evaluating the performance of a model [40]. It plots the trade-off between the true positive rate and the false positive rate for different thresholds of a model. Although it is meant for binary classification, it can be expanded to be used for multi-class classification by binarizing the output and computing the ROC curve per category. However, ROC curves are affected by unbalanced datasets and can result in incorrect interpretations. Fortunately, there is a similar metric that can be used for unbalanced datasets known as precision-recall curves. It is calculated by comparing trade-off between the precision and recall at different thresholds instead of comparing the true positive rate (recall) and false positive rate. 

**Figure 10:**
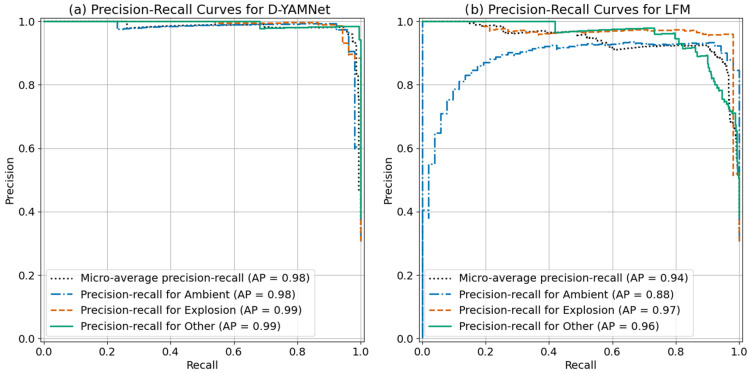
The precision-recall curves for (**a**) D-YAMNet and (**b**) LFM.

Since the dataset used for training the models were greatly unbalanced, we calculated the precision-recall curves including the sample weights to compare and analyze the performance of D-YAMNet and LFM as seen in Figure 10. From initial glance of the two graphs, we see that both D-YAMNet and LFM performed well, D-YAMNet performed significantly better showing a precision-recall graph of a nearly perfect classifier. D-YAMNet (Figure 10a) performed especially well in the “other” category, maintaining a high precision with the increase in recall, which follows our initial analysis of the model. Similarly, the precision-recall curves for LFM follows our initial assessment. We see that both “explosion” and “ambient” maintain high precision at high recall, with the “other” category performing worse. Overall, the introduced models performed well at classifying the three categories, with D-YAMNet being the better classifier. This could possibly be due to the benefits of transfer learning, which can minimize the effects of the small dataset size. 

### 4.2. Cross-Validation of D-YAMNet and LFM

Although the initial assessment on the performance of D-YAMNet and LFM seem promising and explainable, it is still crucial to investigate the models’ consistency and reliability as the overall size of the dataset is small and is susceptible to overfitting and sampling bias. A common method to evaluate the produced model’s performance is through cross-validation. This method works by sub-dividing the dataset into fixed number of subsets. Then, one of the subset is used as the test set and the rest for training a model. This process is repeated so that each subset is used as a test set. Finally, the result from each model is averaged to find a more robust performance estimate.

Since we are investigating the performance of the introduced models (D-YAMNet & LFM), it is important to match the distribution of the dataset used for training and testing for the cross-validation. To accomplish this, we employed a stratified group k fold with k being 5 to sub-divide the dataset. This ensures that the distribution of the categories will be similar and the “ambient” and “explosion” data would be split by explosion event. Additionally, Dividing the data into 5 subsets allows for training the model with roughly a 60%, 20% and 20% for the training, validation, and test sets. However, unlike the original split used to train the introduced models, we were not able to keep a similar explosion characteristic distribution between the training and test set due to the limited number of explosion events with similar yield categories as seen in Figure 3b. 

**Figure 11:**
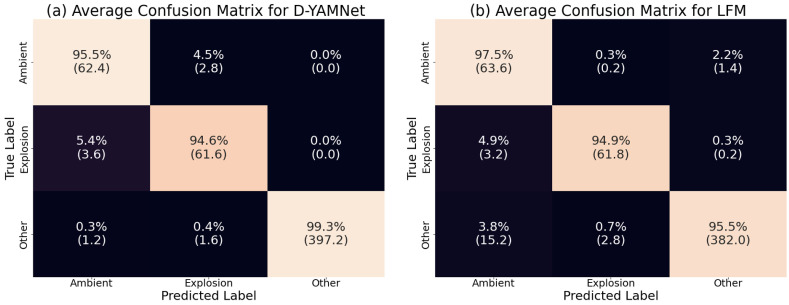
The average confusion matrix for (**a**) D-YAMNet and (**b**) LFM.

The results of the cross-validation for both D-YAMNet and LFM are presented by averaging each subset’s confusion matrix as seen in Figure 11. At initial glance, we see a similar trend to the confusion matrices of the introduced models. The average confusion matrix for D-YAMNet (Figure 11a) performs well in the “other” category and worse in the “ambient” and “explosion” categories. Similarly, the average confusion matrix for LFM (Figure 11b) performs better in the “ambient” and “explosion” categories and worse in the “other” category. However, there is a notable difference compared to the introduced models. There is a significant increase in “explosion” data being predicted as “ambient”, affecting the recall of the “explosion” category by around 3.3% for both models. Upon inspecting the individual confusion matrix for the subsets, we saw that this increase was related to difference in yield distribution of the explosion events between the training and test sets. The subsets with similar yield distributions (i.e. Figure 3b) performed near identical to the introduced models, however as the yield distributions became dissimilar (underrepresented or missing yield categories), the “explosion” category performed worse. This worse performance is expected as overfitting is likely to happen when there are discrepancies in data distribution between the training and test sets. 

Overall, we saw that the cross-validation produced similar results for both D-YAMNet and LFM except for the increased false negatives for the “explosion” category due to the difference in yield distribution between the training and test set. However, this also shows the limitations of the model for deployment as the distribution of explosion yield may not match what is observed, highlighting the need for more robust explosion data.

### 4.3. Model Performance on Longer-Duration Data

To accurately assess the suitability of the model for persistent monitoring, additional, longer-duration data are necessary. To gain some insight into the model’s performance on longer time scales, 10 min of data recorded by two smartphones during an explosion event in the test set were selected at random. The event that was selected was ““INL_20220714_07” and included two smartphones with IDs “180616311” (phone 18) and “2122963039” (phone 21). The effective yield of the explosion was 45.4 kg, and the smartphones were 621 m (phone 18) and 1984 m (phone 21) from the explosion. The 10 min of data were windowed into 0.96 s bins with 50% overlap, totaling 1247 predictions per smartphone data.

**Figure 12:**
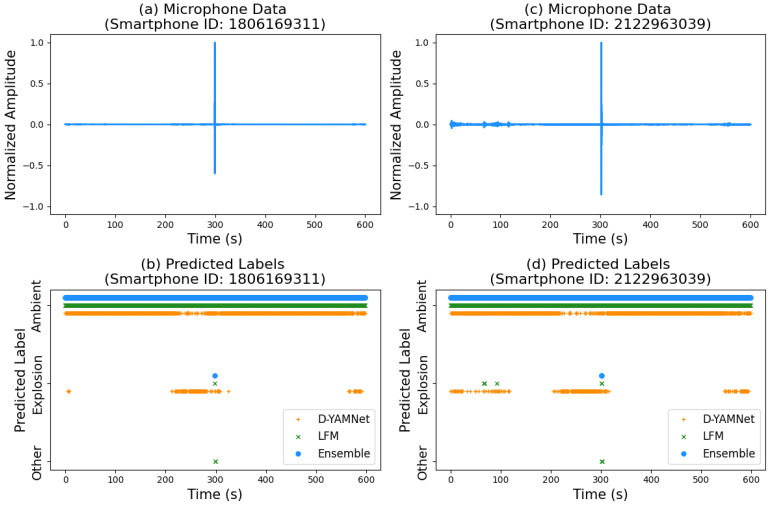
Expanded normalized microphone data from SHAReD for event INL_20220714_07 for smartphone ID (**a**) 1806169311 and (**c**) 2122963039 and the corresponding predicted labels from D-YAMNet, LFM, and the ensemble model for smartphone ID (**b**) 1806169311 and (**d**) 2122963039. The predicted labels were obtained on segmented section of the full waveform with 0.96 s duration and 50% overlap.

The acoustic data of the two smartphones along with the predicted labels from each model (D-YAMNet, LFM, and Ensemble) are shown in Figure 12. From observation of the normalized amplitude data in Figure 12a,c, we see that the data have very little noise, with amplitudes much, much lower than the amplitude of the explosion signals. This is due to the nature of explosion experiments, which, by necessity, are almost exclusively conducted in remote areas with relatively low activity and thus few sources of anthropogenic noise. This is important to keep in mind when accessing model performance.

In Figure 12b,d, we see that the ensemble model successfully detected the explosion for both phones (one count for phone 18, two counts for phone 21). D-YAMNet’s performance is characterized by a high number of false positive cases for “explosion” with counts of 145 and 238 for phones 18 and 21, respectively. This is unsurprising as D-YAMNet is known to have difficulty differentiating “explosion” and “ambient” signals. In contrast, LFM performed well with zero (phone 18) and three (phone 21) false positive cases for “explosion”. Interestingly, there were two false positives for “other” immediately following the correctly detected explosion for both phones. This may be due to remnants of the explosion pulse, as the models were trained on the onset of the explosion and did not always include the full pulse due to the short duration of signals in the dataset. Further investigation of signal duration and model performance could prove fruitful; however, that is beyond the scope of this paper.

Due to the limited duration and variety of explosion data available, this investigation of model performance still falls “short” in fully assessing the suitability of the model for persistent monitoring. However, the ensemble model shows clearly improved performance over D-YAMNet and LFM in the context of deployments in remote areas, and it is especially (and importantly for persistent monitoring) effective at reducing false positives. Further investigation of model performance with longer duration data collected in more “noisy” environments will be planned for future works.

### 4.4. Baseline Model Comparison

Since explosion detection on smartphone sensors using machine learning is a relatively new endeavor, there are no specialized explosion detection models using smartphone data that can be used as a baseline comparison to evaluate the ensemble model’s performance on the three categories. Although there are models that relate to explosion detection [41,42,43,44], the difference in required input data or need of specific calibration make a one-to-one comparison difficult. However, it is useful to compare to similar models to provide a relative performance metric for the introduced models. 

**Figure 13:**
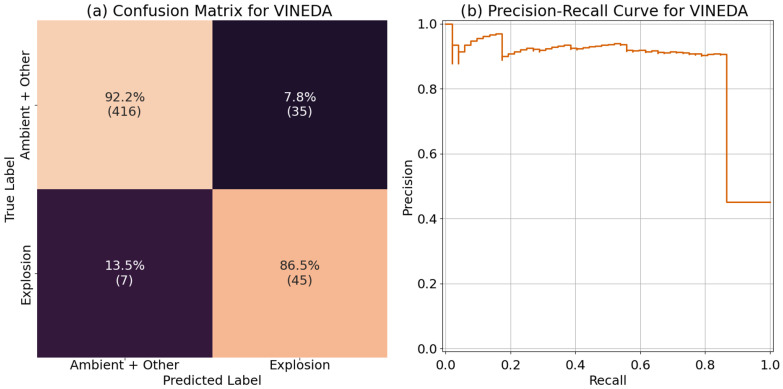
The (**a**) confusion matrix and (**b**) precision-recall curves for the YAMNet model. The average precision (AP) for VINEDA was 0.86.

For this comparison we chose the Volcanic INfrasound Explosions Detector Algorithm (VINEDA), which was developed for the automated explosion detection of volcanic explosions from acoustic infrasound data [41]. This algorithm works by taking the acoustic data and returning a characteristic function (CF) that can be used for detecting explosions. There are multiple adjustable parameters for the algorithm that relate to the frequency range, duration, and shape of the explosion pulse. The parameters were chosen by iterating through different values on the training and validation set and selecting the best performing set which was the following: $f_{low}=1,\;f_{high}=100,\;N_{fb}=10,\;D_{min}=0.1,\;D_{max}=0.3,\;N_{db}=3,\;\beta =2$. The confusion matrix and precision-recall curve for the test set is shown in Figure 13. Since VINEDA is a detector for explosions and does not include a ”other” category, the “ambient” and “other” category were consolidated, and the model is evaluated as a binary classifier for explosions. The confusion matrix (Figure 13a) was calculated by classifying data that had a maximum value for CF greater than 0 as an explosion. Overall, VINEDA performed decent with recall of 92.2% for the “ambient + other” category and 86.5% for the “explosion” category. Upon further examination of the false positives in the “explosion” category, we found that most were from ESC-50 data that included low and/or infrasound frequency. For the precision-recall curve (Figure 13b), we see that the classifier does a good job at keeping high precision with increased recall until reaching around 85% recall where the precision suddenly drops. Comparing this to the precision-recall curves of D-YAMNet and LFM (Figure 10a,b), we see that although VINEDA performs well, both D-YAMNet and LFM performed better. However, it is still important to note that VINEDA performs well considering that the explosion data being used for this comparison has a diminished frequency response in the infrasound frequencies that would affect the VINEDA algorithm.

**Figure 14:**
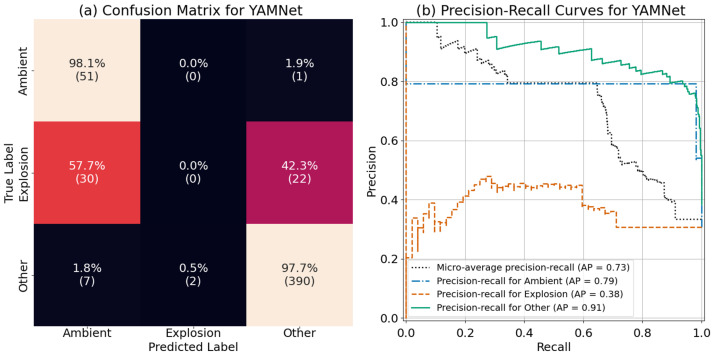
The (**a**) confusion matrix and (**b**) precision-recall curves for the YAMNet model.

Additionally, the base YAMNet model does include an “explosion” class that can be used for a comparison. We used the test dataset to evaluate the performance of YAMNet by mapping the 521 classes as follows: “explosion” if YAMNet predicted “explosion”, “ambient” if YAMNet predicted “silence”, and “other” for all other classes. The resulting confusion matrix and precision-recall curves are shown in Figure 14. Examining the confusion matrix (Figure 14a), we see that YAMNet performed well at classifying “ambient” and “other” sounds, with 98.1% and 97.7% recall, respectively. However, it was not able to correctly classify a single explosion properly. More interestingly, it seems to have categorized roughly half of the “explosion” sounds as “ambient” and half as “other”. Looking at the precision-recall curve (Figure 14b), we can confirm our observations from the confusion matrix results. YAMNet performs well in distinguishing the “other” and “ambient” category, and poorly in the “explosion” category. This poor performance of the base YAMNet model is most likely due to the “explosion” category in the original YAMNet training data (AudioSet), which mostly consists of “explosion sounds” from video games and movies, as well as gunshots, which aren’t HEs.

### 4.5. Future Work

Although the initial success of the ensemble model is promising, it is still based on a limited dataset (<3000 waveforms). Continued efforts in explosion data collection and public release of such data will be essential for improving the performance of explosion detection models. Future work should include deploying smartphones to test the ensemble model in deployments for real-time detection and gather performance data in a persistent monitoring setting. Additionally, developing algorithms to take into account the explosion detection results from all nearby smartphones and/or incorporating other algorithms designed to detect explosions should improve reliability. It would also be ideal to replace the “other” data with data recorded on smartphones, making the recording instrument consistent between all training data. The results of the ensemble model presented in this work indicate that, with more data to train models on, rigorous testing in the field, and effective integration of predictions from multiple models and devices, smartphones will prove to be a viable ubiquitous sensor network for explosion detection and a valuable addition to the arsenal of explosion monitoring. The data and models presented in this paper are available to the public [19] and we encourage anyone interested in explosion detection models to replicate, expand upon, and/or improve the results presented in this work.

## Data Availability

The data (SHAReD and expanded dataset [19]) used for this research are available as a pandas DataFrame [45] along with D-YAMNet and LFM. The data and models can be found in the Harvard Dataverse open access repository with the following Digital Object Identifier doi: 10.7910/DVN/ROWODP.
